# Supplementation-Dependent Effects of Vegetable Oils with Varying Fatty Acid Compositions on Anthropometric and Biochemical Parameters in Obese Women

**DOI:** 10.3390/nu10070932

**Published:** 2018-07-20

**Authors:** Luciene Oliveira-de-Lira, Eduila Maria Couto Santos, Raphael Fabrício de Souza, Rhowena Jane Barbosa Matos, Matilde Cesiana da Silva, Lisiane dos Santos Oliveira, Taís Galdêncio do Nascimento, Paulo Artur de Lara Schinda Schemly, Sandra Lopes de Souza

**Affiliations:** 1 PostGraduate Program, Department of Nutrition, Federal University of Pernambuco, Pernambuco 50670-901, Brazil; tais_galdencio96@hotmail.com (T.G.d.N.); sanlopesufpe@gmail.com (S.L.d.S.); 2 Academic Center of Vitoria de Santo Antão, Federal University of Pernambuco, Pernambuco 55608-680, Brazil; eduila@hotmail.com (E.M.C.S.); rhowenajane@gmail.com (R.J.B.M.); matildecesiana@hotmail.com (M.C.d.S.); lisianenutricao@yahoo.com.br (L.d.S.O.); 3 Department of Physical Education, Federal University of Sergipe—UFS, São Cristovão, Sergipe 49100-000, Brazil; raphaelctba20@hotmail.com (R.F.d.S.); pshinda@bol.com.br (P.A.d.L.S.S.)

**Keywords:** obesity treatment, coconut oil, sunflower oil, chia oil, dietary re-education, lipid and glycemic profile

## Abstract

Fatty acid (FA) composition is a determinant of the physiological effects of dietary oils. This study investigated the effects of vegetable oil supplementation with different FA compositions on anthropometric and biochemical parameters in obese women on a hypocaloric diet with lifestyle modifications. Seventy-five women (body mass index, BMI, 30–39.9kg/m^2^) were randomized based on 8-week oil supplementation into four experimental groups: the coconut oil group (CoG, *n* = 18), the safflower oil group (SafG, *n* = 19), the chia oil group (ChG, *n* = 19), and the soybean oil placebo group (PG, *n* = 19). Pre- and post-supplementation weight, anthropometric parameters, and body fat (%BF), and lean mass percentages (%LM) were evaluated, along with biochemical parameters related to lipid and glycidemic profiles. In the anthropometric evaluation, the CoG showed greater weight loss (Δ% = −8.54 ± 2.38), and reduced BMI (absolute variation, Δabs = −2.86 ± 0.79), waist circumference (Δabs = −6.61 ± 0.85), waist-to-height ratio (Δabs = −0.041 ± 0.006), conicity index (Δabs = −0.03 ± 0.016), and %BF (Δabs = −2.78 ± 0.46), but increased %LM (Δabs = 2.61 ± 1.40) (*p* < 0.001). Moreover, the CoG showed a higher reduction in biochemical parameters of glycemia (Δabs = −24.71 ± 8.13) and glycated hemoglobin (Δabs = −0.86 ± 0.28) (*p* < 0.001). The ChG showed a higher reduction in cholesterol (Δabs = −45.36 ± 0.94), low-density lipoprotein cholesterol (LDLc; Δabs = −42.53 ± 22.65), and triglycerides (Δabs = −49.74 ± 26.3), but an increase in high-density lipoprotein cholesterol (HDLc; abs = 3.73 ± 1.24, *p* = 0.007). Coconut oil had a more pronounced effect on abdominal adiposity and glycidic profile, whereas chia oil had a higher effect on improving the lipid profile. Indeed, supplementation with different fatty acid compositions resulted in specific responses.

## 1. Introduction

Obesity is a chronic disease of multifactorial origin arising from a long-term energy imbalance. It is characterized by fat accumulation in adipose tissues that can cause serious health problems and is often associated with comorbidities such as glucose and lipid metabolism disorders [1]. It is one of the main chronic diseases worldwide, with increasing epidemiological trends. The World Health Organization estimates that by 2025, a total of 700 million people (i.e., 5.4% of the world’s population) will suffer from obesity [2]. Brazil also has an increasing prevalence of obesity (20.8% of the population is affected), and it predominantly occurs in women (24.4% women vs. 16.8% men) [3].

Treatments that are currently available have failed to prevent an increase in obesity prevalence [4]. Diet therapy and increased physical activity (PA) [5] reportedly do not result in significant weight loss in the short term, resulting in low adherence to treatment [4]. In contrast, current anti-obesity drugs and surgical treatments have side effects that sometimes outweigh their benefits [6]. Therefore, novel, effective, and safe strategies that are adjuvants to current treatment methods are required. Among these, use of oils with specific fatty acids (FAs) such as medium-chain FAs (MCFAs) and mono- and polyunsaturated FAs has demonstrated anti-obesogenic effects [7,8]. Some sources of these FAs include coconut oil (*Cocos nucifera* L.), a source of MCFAs [9]; safflower oil (*Carthamus tinctorius*), a source of linoleic FA (55–88%) [10]; and chia oil (*Salvia hispanica* L.), which contains approximately 60% α-linolenic acid (ALA) [11].

Consumption of the aforementioned oils in the form of capsules has become common for the purpose of weight loss, and they are often used indiscriminately and without nutritional guidance. Capsules are also used in studies for evaluating the physiological effects of specific FAs. In addition to being practical, they mask the taste of oils, protect FAs against oxidation, and make supplementation standardization easier in studies, eliminating possible conformational chemical changes resulting from cooking processes [12].

The physiological role of lipid intake is often a point in question, given its potential beneficial or harmful effects depending on fatty acid composition. Most of the fatty acids present in vegetable oils are associated with health benefits [13,14]. However, the effect of vegetable oil supplementation as an adjuvant to the obesity nutritional therapy, particularly in the form of capsules, remains uncertain, with few studies and conflicting results. The determination of the real effects on obesity and comorbidities is necessary for the proper nutritional management of these oils. Here, we aimed to evaluate the supplementation effects of safflower, coconut, and chia oils on anthropometric and biochemical parameters in women with obesity under a hypocaloric diet with standard lifestyle modifications, namely dietary re-education and PA stimulation.

## 2. Methods

### 2.1. Participants

We included women aged between 20 and 40 years who were not menopausal, had a body mass index (BMI) ≥30 kg/m^2^ and ≤39.9 kg/m^2^, and waist circumference (WC) of >88 cm [15], characteristic of abdominal adiposity. They were recruited from the Reference Center on Women’s Health, Moreno City, Pernambuco, Brazil. The study protocol was approved by the Research Ethics Committee of the Health Sciences Center of the Federal University of Pernambuco (CAAE 62661416.9.0000.5208) and registered at ClinicalTrials.gov (RBR-36bjsc). All participants signed a written informed consent form. The procedures adopted are in accordance with the Declaration of Helsinki of the World Medical Association.

The exclusion criteria were as follows: pregnancy or smoking; history of cardiovascular, hepatic, renal, neurological, hematological/oncological, endocrine, or gastrointestinal disease; history of eating or psychiatric disorders; allergic reactions; or use of drugs that could interfere with the lipid or glycemic profile.

### 2.2. Study Design and Nutrition Intervention

**Figure 1:**
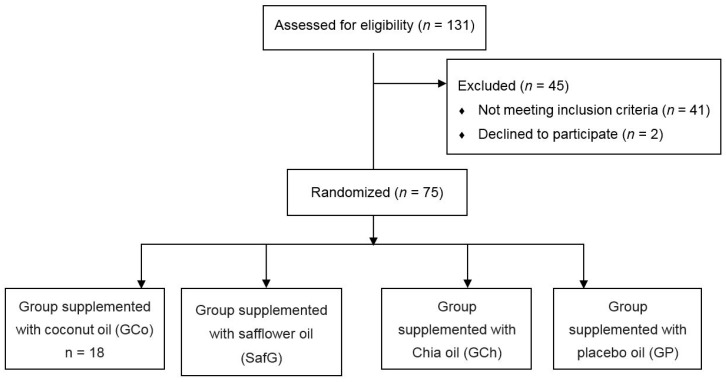
CONSORT diagram of participants representing the experimental groups. Anthropometric assessments, biochemical blood profile, estimated dietary intake, and subjective physical activity were evaluated one week before and one week after the end of the supplementation protocol.

A randomized, double-blind, placebo-controlled clinical trial was conducted. Seventy-five participants were randomized and BMI-matched for 8-week oil supplementation into four experimental groups: the coconut oil group (CoG, *n* = 18), the safflower oil group (SafG, *n* = 19), the chia oil group (ChG, *n* = 19), and the soybean oil group (placebo group, PG, *n* = 19). The latter was chosen as the placebo considering that it is used by the majority of the Brazilian population in culinary preparations. A flow chart representing the experimental protocol is presented in Figure 1.

The oils were offered in the form of capsules of similar color, flavor and form, and were supplied by Mosteiro Dévakan Ltd. (São Paulo, Brazil). To ensure quality, centesimal composition analysis of the main FAs was performed using high-performance liquid chromatography in the Food Analysis Laboratory of the Department of Nutrition at the Federal University of Pernambuco (for more details about centesimal composition oils, refer to Supplementary Material (Table S1). All participants were instructed to ingest two capsules (1 g/capsule) 30 min before main meals (6 g/day).

The capsules were filled by a pharmacist who was not affiliated to our research group, in plastic containers with different-colored lids; this codified the oils. The investigators and participants were blinded to the allocation of treatment. The significance of the lid colors was revealed only after the data were collected and analyzed.

The participants were instructed to follow a balanced diet according to the recommendations of Brazilian Association for the Study of Obesity and Metabolic Syndrome—ABESO [5]. They all received guidance from the same nutritionist. An average of 500 kcal of the usual individual energy intake was subtracted, which was evaluated using a three-day food application. In addition, they were advised to perform regular PA in the form of walking for a minimum of 50 min four times a week [5]. The effectiveness of diet adherence was evaluated through changes in anthropometric parameters at the time of biweekly visits to the nutritionist, during which the oils were distributed and PA stimulation was reinforced.

### 2.3. Assessments

Data on age, schooling, and socioeconomic status [16] were collected at the time of recruitment. Data on anthropometric and biochemical parameters, energy intake, and PA subjective assessment [17] were collected one week before the start of dietary intervention and supplementation (T1) and one week after the end of the study (T2).

### 2.4. Body Composition

Weight, height, and WC were evaluated. From these data, BMI, waist-to-height ratio (WHR) [18], and conicity index (CI) were calculated [19]. The percentages of body fat (%BF), lean mass (%LM), and water (%water) were obtained by bioimpedance. In addition, we evaluated the percentage of weight loss, considering a clinically significant loss of >5% and/or 10% [20].

### 2.5. Biochemical Evaluation

Blood samples were collected in the morning after fasting overnight for 8–12 h in order to quantify total cholesterol, low- and high-density lipoprotein cholesterol (LDLc and HDLc, respectively), triglycerides (Tg), and glycosylated hemoglobin (HbA1c) levels. Mean estimated glycemia (MEG) was obtained using a specific formula from HbA1c (MEG = 28.7 × HbA1c − 46.7) [21]. From the obtained results, the Tg/HDL ratio [22] was calculated. A detailed description of anthropometric and biochemical parameters is available in Supplementary Material (Table S2).

## 3. Statistical Analysis

Normality was verified through the Shapiro–Wilk test and the equality of variances through the Levene F test. For parametric data, the paired t-test was used in the comparison between T1 and T2 times, followed by analysis of variance (two-way ANOVA) with post hoc Tukey´s test. For the non-parametric data, the Kruskal–Wallis and Wilcoxon paired tests were used. For evaluation of the size, Cohen’s d was used. Pearson’s Chi-square test or Fisher’s exact test was used when comparing the groups for categorical variables when the condition for Chi-square use was not verified. Statistical analysis was performed using SPSS software (version 23.0^®^) and MedCalc version 14.8.1. A significance level of 5% (*p* < 0.05) was utilized.

## 4. Results

It is noteworthy that 70.7% participants were in social classes B2 and C1, 69.3% were married, and 73.3% stated that they had completed high school. The mean age of the study population was 34.07 years (CoG = 33.17 ± 5.11, SafG = 35.58 ± 5.23, ChG = 35.00 ± 5.29, and PG = 31.58 ± 5.85 years; *p* > 0.05). Socioeconomic data are presented in Table 1.

**Figure 2:**
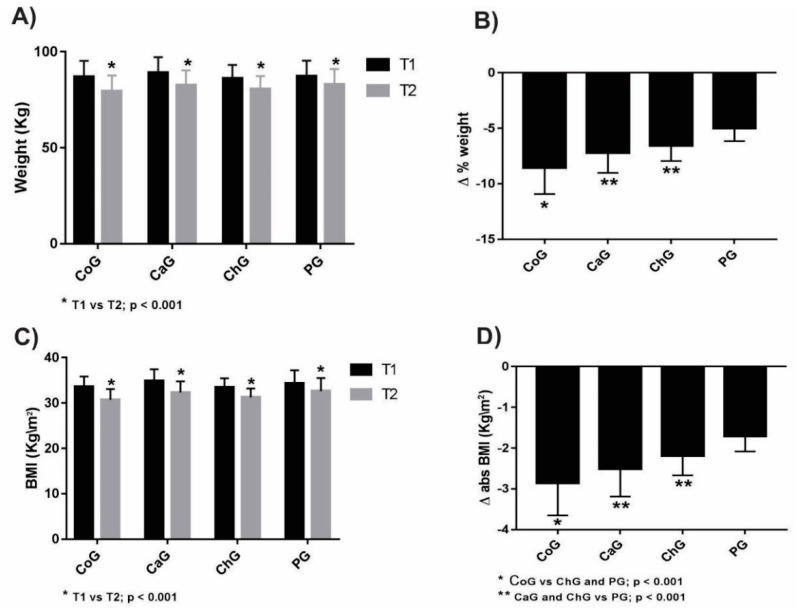
Variation of weight loss and body mass index (BMI). Percentage weight loss (**A**) and absolute BMI loss (**C**) (T1 vs. T2). Absolute weight variation (**B**) and BMI variation (**D**). Results presented in mean + standard deviation. T1 = Value one week before (T1) and one week after (T2) supplementation protocol; Δ = T2 − T1. CoG—Group supplemented with coconut oil; SafG—Group supplemented with safflower oil; ChG—Group supplemented with chia oil; PG—Group supplemented with soybean (placebo) oil.

Weight loss (T1 vs. T2) was observed in all groups (*p* < 0.001, Figure 2A). The CoG showed a higher variation between the times (Δ% = −8.54 ± 2.38, *p* < 0.001, Cohen’s d = 0.89) than the ChG (Δ% = −6.54 ± 1.41, Cohen’s d = 0.81) and PG (Δ% = −5.00 ± 1.16, Cohen’s d = 0.53). The SafG (Δ% = −7.19 ± 1.83, Cohen’s d = 0.81) and ChG showed similar variations, being superior to the PG (*p* < 0.001) (Figure 2B).

BMI showed a similar trend to weight. All groups showed a reduction in relation to absolute variation (Δabs) (T1 vs. T2, *p* < 0.001, Figure 2C). The CoG showed a more pronounced variation between the times (Δabs = −2.86 ± 0.79, *p* < 0.001, Cohen’s d = 1.25) than the ChG (Δabs = −2.19 ± 0.48, Cohen’s d = 1.67) and the PG (Δabs = −1.71 ± 0.37, Cohen’s d = 0.57). The SafG (Δabs = −2.51 ± 0.68, Cohen’s d = 0.99) and ChG showed similar and larger variations than those in the PG (*p* < 0.001, Figure 2D). Regarding clinical significance, a higher percentage of women had weight loss of ≥5% (94.4%) and 10% (22.2%) in the CoG. Table 2 presents the classification of percentage change in weight loss.

**Figure 3:**
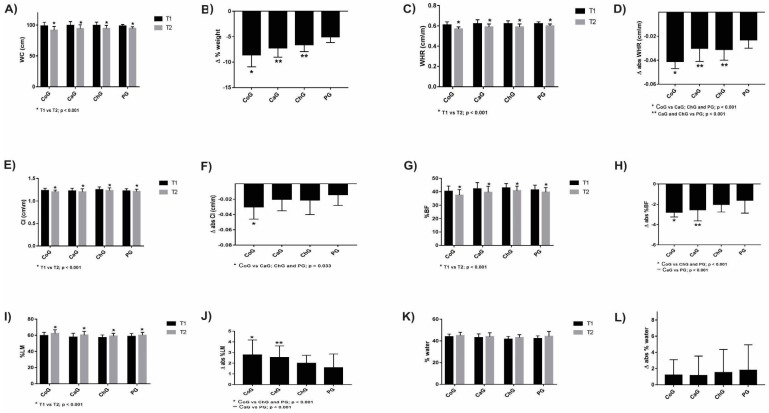
Anthropometric characteristics of participants before (T1) and after (T2) supplementation.T1 vs. T2: WC (**A**), WHR (**C**), CI (**E**),%BF (**G**), %LM (**I**), and %water (**K**). Absolute variation (T1 vs. T2) of WC (**B**), WHR (**D**), CI (**F**), %BF (**H**),%LM (**J**), and %water (**L**). WC—waist circumference; WHR—waist-to-height ratio; CI—conicity index; %BF—body fat percentage; %LM—lean mass percentage; %water—percentage of hydration; CoG—Group supplemented with coconut oil; SafG—Group supplemented with safflower oil; ChG—Group supplemented with chia oil; PG—Group supplemented with soybean (placebo) oil. Results presented as mean + standard deviation (SD).

A reduction in the anthropometric parameters (T1 vs. T2) WC, WHR, CI, and %BF (Figure 3A,C,E,G) and an increase in %LM (Figure 3I) were observed in all experimental groups (*p* < 0.001). The CoG, however, showed higher variations in WC (Δabs = −6.61 ± 0.85, *p* < 0.001, Cohen’s d = 1.09), WHR (Δabs = −0.041 ± 0.006, *p* < 0.001, Cohen’s d = 1.33), and CI (Δabs = −0.03 ± 0.016, *p* < 0.001, Cohen’s d = 0.76) relative to the SafG, ChG, and PG.

A higher reduction in %BF was observed in the CoG (Δabs = −2.78 ± 0.46, *p* < 0.001, Cohen’s d = 0.89) than in the ChG and PG, whereas a higher reduction in %BF was achieved in the SafG than in the PG (Δabs = −2.53 ± 1.09, *p* < 0.001, Cohen’s d = 0.79). The %LM increase was higher in the CoG (Δabs = 2.61 ± 1.40, *p* < 0.001, Cohen’s d = 0.71) than in the ChG and PG. The %LM increase in the SafG was higher than that in the PG.

The CoG showed higher Δabs for HbA1c (Δabs = −0.86 ± 0.28, *p* < 0.001) relative to the SafG (Δabs = −0.49 ± 0.26, *p* < 0.001, Cohen’s d = 1.28), ChG (Δabs = −0.53 ± 0.21, *p* < 0.001, Cohen’s d = 1.83), and PG (Δabs = −0.36 ± 0.18, *p* < 0.001, Cohen’s d = 1.05); Δabs to MEG was also higher in the CoG (Δabs = −24.71 ± 8.13, *p* < 0.001, Cohen’s d = 1.65) in relation to the SafG (Δabs = −14.20 ± 7.44, *p* < 0.001, Cohen’s d = 1.28), ChG (Δabs = −15.26 ± 5.98, *p* < 0.001, Cohen’s d = 1.84), and PG (Δabs = −10.42 ± 5.17, *p* < 0.001, Cohen’s d = 1.03). Relevant data are presented in Table 3.

There was a reduction in the total caloric intake (T1 vs. T2) in all experimental groups (*p* < 0.05). Energy variation in the CoG (Δabs = −612.69 ± 0.81.2, *p* < 0.016, Cohen’s d = 5.81) was higher than in SafG (Δabs = −500.49 ± 73.54, *p* < 0.016, Cohen’s d = 1.81), ChG (Δabs = −532.59 ± 75.38, *p* < 0.016, Cohen’s d *=* 1.20), and PG (Δabs = −532.50 ± 89.84, *p* < 0.016, Cohen’s d = 1.92) (Table 4). At the end of the study, all groups showed an increase in the percentage of PA levels (T1 vs. T2), with no significant difference among the groups (Table 5).

## 5. Discussion

Eight weeks of daily supplementation were sufficient to observe different supplementation-dependent effects on anthropometric and biochemical parameters according to the different oils used in the present study when this was associated with a hypocaloric diet and lifestyle modifications. With regard to coconut oil supplementation, we observed a higher adjuvant effect on weight loss and %BF, with an emphasis on the reduction of anthropometric parameters associated with abdominal adiposity. Our findings are consistent with those reported by Assunção et al. [23] and Cardoso et al. [24], who reported a reduction in WC that was associated with a significant increase in HDLc after coconut oil supplementation as compared to placebo oil (a source of long-chain FAs (LCFAs)) supplementation in obese women. Our findings regarding the reduction in WHR and CI reinforce the potential effects of coconut oil in causing a decrease in abdominal adiposity.

Unlike LCFAs, MCFAs do not require chylomicron transport via the lymphatic system to reach their target tissues, thereby favoring hepatic metabolism and mitochondrial oxidation [25,26]. These FAs would still be favored because they do not depend on carnitine palmitoyltransferase-1, which is a key enzyme facilitating the entry of FAs into the mitochondria for further oxidation [26]. Therefore, the intake of oils containing MCFA should result in increased thermogenesis and fat oxidation with a consequent loss of fat mass, potentially aiding in stimulating weight loss [26].

Because coconut oil is a source of saturated FAs, an important reason behind the debate involves the possible adverse effects of coconut oil on the lipid profile and consequent risk of cardiovascular disease. Although the literature has reported conflicting results [23,24,27], women supplemented with coconut oil did not show any adverse changes in their lipid profile in this present study. On the contrary, they experienced beneficial effects, such as an increased HDLc levels and decreased Tg/HDL ratio as compared to the PG.

Our findings are in agreement with those of Assunção et al. [23], Cardoso et al. [24], and Chinwong et al. [28], who reported no deleterious effects and increased HDLc levels in women that took coconut oil supplements. The epidemiological evidence from the populations consuming substantial amounts of coconut oil reinforces the idea of the lack of negative effects on cardiovascular health [29]. In a systematic review, Eryres et al. [25] did not find evidence of any adverse effects of consuming coconut oil on the lipid profile. Furthermore, in a 28-day study involving postmenopausal women without nutritional monitoring, Harrys et al. [27] compared the impact of coconut oil supplementation with that of safflower oil supplementation and reported that the former significantly increased TC LDLc, and HDLc levels and decreased Tg levels. Therefore, the inconsistencies observed between studies are justified by the nutritionally different context in which they are performed and the period during which supplementation is introduced.

The positive effect of coconut oil supplementation on the glycemic profile in the present study is consistent with the findings from studies in rats [30,31], which reported an improvement in glycemia and insulin response in individuals with obesity when supplemented with MCFAs. This could be due to the effect of polyphenolic anti-oxidants in coconut oil, such as caffeic acid, ferulic acid, syringic acid, catechin, and epigallocatechin, which are known to have anti-diabetic and insulin-sensitizing effects [32,33]. The glycemic response may have also contributed to the higher weight loss observed in the CoG, with weight loss also improving glycemic parameters. Thus, this generates positive feedback.

Although the total caloric intake was similar among the groups, the higher observed decrease in the CoG may have contributed to the higher weight loss observed. As a source of fat during breakfast, coconut oil has been reported to increase satiety in obesity woman [34]. However, Kingela et al. [35] did not report such an increase upon the use of coconut oil compared to isolated MCFAs. Deol et al. observed more obesogenic and diabetogenic effects when using soybean oil compared to coconut oil in mice [36].

The SafG and (to a lesser degree) the ChG showed beneficial effects in relation to weight loss. These effects could be because polyunsaturated LCFAs, such as linoleic and linolenic acids, play important roles as peroxisome proliferator-activated receptor α ligands, inhibiting the differentiation of preadipocytes into mature adipose tissue cells. Polyunsaturated LCFAs also suppress the activity of sterol regulatory element-binding protein-1c and the expression of carbohydrate response element-binding protein, which are transcription factors that induce lipogenesis. This potentially implies a gene expression profile that favors lipolysis [7,8].

In its metabolic pathway, linoleic acid is converted to arachidonic acid (AA), which has contradictory roles in relation to adipogenesis. According to Madsen et al. [37], such contradictions result from the fact that the physiological effect of AA on adipocyte differentiation is related to cAMP signaling, which plays a key role in controlling prostaglandin production. A high glucagon/insulin ratio, which was caused by a decrease in carbohydrate supply and an increase in protein intake observed in the study population, improves cAMP-dependent signaling pathways, resulting in the production of anti-adipogenic prostaglandins that function in the adaptive reactions of cyclooxygenases. In this context, AA action would result in increased lipolysis of white adipose tissue. Thus, the dietary context in which supplementation with safflower oil was introduced may be a determinant for AA action [37]. AA was still associated with the browning of adipose tissue in rats, which protects against the deleterious effects of obesity [38]. In addition, the active components of safflower oil may inhibit preadipocyte proliferation and adipogenesis in rats, potentiating the expression of hormone-sensitive lipase and inhibiting the action of lipoprotein lipase. This results in a lipolytic state that favors weight loss [39,40].

In this study, the ChG showed predominance over other groups with respect to a decrease in CT, LDLc, and Tg levels and an increase in the HDLc level compared to that in the PG, which contributes to a decrease in cardiovascular risk. The type of dietary FA may modulate the amount of intracellular plasma cholesterol. During its metabolic pathway, the ALA present in chia oil is converted to eicosapentaenoic acid (EPA, C22:5) and docosahexaenoic acid (DHA, C22:6) [41]. EPA and DHA are associated with beneficial changes in lipid metabolism, which may alter serum cholesterol concentrations by reducing Tg levels and increasing plasma HDLc levels [7,8,11,40,42].

Few studies have evaluated the specific effects of chia oil supplementation as a source of ALA on biochemical parameters, particularly in humans. Consistent with our results, Santos-Lopez et al. observed that chia oil supplementation in elderly rats reduced cholesterol [43]. In a cross-sectional study conducted by Dittrich et al. involving humans with hypertriglyceridemia, a decrease in Tg levels was reported after supplementation of oil containing ALA [44]. Similarly, Mirfatabi et al. [45] reported a reduction in serum Tg levels in hemodialysis patients who consumed flaxseed oil, which is another important source of ALA. In addition, Zhao et al. [46] reported a decrease in LDLc, HDLc, and Tg levels, with increased dietary amounts of ALA in a study involving hypercholesterolemic men. In a recent meta-analysis, Maki and Dicklin found evidence supporting the beneficial effects of consuming chia oil in terms of the lipid profile [47].

Dietary re-education and lifestyle modifications are important for anti-obesity treatment, and should lead to an improvement in anthropometric and biochemical parameters [48]. Nutritional orientation, resulting in changes in dietary habits, was defined by increased protein and fiber intake but decreased carbohydrate intake in addition to an increase in PA, possibly playing a role in determining the results obtained in this study.

In conclusion, our findings suggest that there are beneficial supplementation-dependent effects of vegetable oils with varying fatty acid compositions on anthropometric and biochemical parameters in obese women who also changed to a hypocaloric diet with further lifestyle modifications. Coconut oil is potentially more effective for treating obesity as it encourages a loss of abdominal adiposity and an improvement in glycemic parameters without any undesirable alterations in the lipid profile, while chia oil seems to have a more pronounced effect in improving lipid parameters. The other biochemical compounds present in the oils may have influenced the results obtained. Nutritional monitoring and PA stimulation were fundamental in obtaining the desired results, contraindicating the current indiscriminate use of supplements without any nutritional guidance. The findings of this study provide support for current recommendations for the use of such supplements in obese people. Further intervention studies with a longer duration need to be conducted in order to better evaluate the effects of supplementation with the above-mentioned oils.

## Figures and Tables

**Table 1:** Socioeconomic characterization of participants.

Variable	*n*	%
Socio economic classification		
B1	7	9.3
B2	17	22.7
C1	36	48.0
C2	15	20.0
Marital status		
Single	13	17.3
Married	52	69.3
Divorced	10	13.3
Degree of schooling		
Primary school	17	22.7
Incomplete primary school	3	4.0
Secondary education	46	61.3
Higher education	6	8.0
Postgraduate	3	4.0

**Table 2:** Percentage of variation in weight loss of the participants.

	Groups								*p*-Value
	CoG (*n* = 18)		SafG (*n* = 19)		ChG (*n* = 19)		PG (*n* = 19)		
	*n*	%	*n*	%	*n*	%	*n*	%	
Weight loss %									
>5%	17	94.4	17	89.5	17	89.5	9	47.4	*p* = 0.002 *
up to 5%	1	5.6	2	10.5	2	10.5	10	52.6	
**Total**	**18**	**100.0**	**19**	**100.0**	**19**	**100.0**	**19**	**100.0**	
Weight loss%									*p* = 0.013 *
>10%	4	22.2	1	5.3	-	-	-	-	
Up to 10%	14	77.8	18	94.7	19	100.0	19	100.0	
**Total**	**18**	**100.0**	**19**	**100.0**	**19**	**100.0**	**19**	**100.0**	

Percentage distribution of women with weight loss considered clinically significant (weight loss greater than 5 or 10%). * = *p* < 0.05%. CoG—Group supplemented with coconut oil; SafG—Group supplemented with safflower oil; ChG—Group supplemented with chia oil; PG—Group supplemented with soybean (placebo) oil.

**Table 3:** Blood profile absolute variation of participants before (T1) and after (T2) the supplementation protocol.

	Groups				*p*-Value
	GCo (*n* = 18)	SafG (*n* = 19)	ChG (*n* = 19)	GP (*n* = 19)	
Biochemical characteristics	Mean ± SD	Mean ± SD	Mean ± SD	Mean ± SD	
Total cholesterol (TC mg/dL)					
T1	215.56 ± 17.85	200.74 ± 25.77	232.47 ± 17.98	214.47 ± 24.05	
T2	198.00 ± 17.60 *	182.95 ± 19.13 *	187.11 ± 17.04 *	195.74 ± 26.22 *	
**Δ**	**−17.56 ± 7.70**	**−17.79 ± 9.10**	**−45.36 ± 8.86 ^a^**	**−18.74 ± 10.27**	***p* < 0.001**
LDLc (mg/dL)					
T1	143.22 ± 18.78	146 ± 26.42	166.16 ± 17.90	142.84 ± 23.74	
T2	128.33 ± 17.72 *	130.63 ± 24.34 *	123.63 ± 18.24 *	127.47 ± 23.21 *	
**Δ**	**−14.89 ± 11.53**	**−15.37 ± 9.35**	**−42.53 ± 22.65 ^a^**	**−15.37 ± 13.78**	***p* < 0.001**
HDLc (mg/dL)					
T1	52.94 ± 7.94	44.53 ± 7.19	45.32 ± 7.17	50.47 ± 7.61	
T2	55.61 ± 6.36 *	47.11 ± 9.82 *	49.05 ± 5.93 *	49.95 ± 7.15	
**Δ**	2.67 ± 158 ^b^	2.58 ± 2.63	3.73 ± 1.24 ^a^	0.52 ± 0.46	***p* < 0.001**
VLDLc (mg/dL)					
T1	21.50 ± 4.26	18.95 ± 5.29	23.47 ± 6.99	21.47 ± 7.41	
T2	17.83 ± 3.17 *	15.74 ± 4.48 *	18.00 ±5.08 *	20.05 ± 8.02 *	
**Δ**	**−3.67 ± 4.56**	**−3.21 ± 4.14**	**−5.47 ± 7.63**	**−1.42 ± 2.76**	***p* = 0.421**
Triglycerides (mg/dL)					
T1	130.89 ± 38.22	129.58 ± 49.34	137.79 ± 36.80	132.47 ± 44.00	
T2	98.33 ± 29.09 *	93.95 ± 36.51 *	88.05 ± 24.42 *	107.53 ± 39.18 *	
**Δ**	**−32.56 ± 24.43**	**−35.63 ± 23.47**	**−49.74 ± 26.36 ^a^**	**−24.94 ± 28.28**	***p* = 0.040**
Tg/HDL rate					
T1	2.52 ± 0.75	3.10 ± 1.77	3.27 ± 1.07	2.70 ± 1.05	
T2	1.78 ± 0.53 *	2.26 ± 1.63 *	1.83 ± 0.58 *	2.24 ± 1.03 *	
**Δ**	**−0.74 ± 0.66**	**−0.84 ± 0.60**	**−1.36 ± 0.88 ^a^**	**−0.46 ± 0.55**	***p* < 0.001**
MEG (mg/dl)					
T1	109.56 ± 7.32	108.28 ± 13.01	110.7 ± 9.53	104.2 ± 11.57	
T2	84.84 ±6.17 *	94.08 ± 8.64 *	95.44 ± 6.79 *	93.78 ± 8.31 *	
**Δ**	**−24.71 ± 8.13** ^c^	**−14.20 ± 7.44 ^d^**	**−15.26 ± 5.98 ^d^**	**−10.42 ± 5.17**	***p* < 0.001**
HbA1C (%)					
T1	5.44 ± 0.25	5.40 ± 0.45	5.48 ± 0.33	5.26 ± 0.40	
T2	4.58 ± 0.21 *	4.91 ± 0.30 *	4.95 ± 0.24 *	4.89 ± 0.29 *	
**Δ**	**−0.86 ± 0.28 ^c^**	**−0.49 ± 0.26**	**−0.53 ± 0.21**	**−0.36 ± 0.18**	***p* < 0.001**

Data given as means and standard deviation (SD). *p* < 0.05 indicates a significant difference. * = T1 vs. T2; a = GCh vs. GCo, GCa, and PL; b = GCo and GCa vs. PL; c = CCo vs. GCh, GCA, and GP; d = GCh and GCa vs. PL. LDLc—low-density lipoprotein cholesterol; HDLc—high-density lipoproteins linked to cholesterol; Tg/HDL—triglyceride/HDL ratio; HbA1c—glycosylated hemoglobin; MEG—mean estimated glycemia; Δ = T2 − T1.

**Table 4:** Food intake variation estimated of participants before (T1) and after (T2) the supplementation protocol.

	Groups				*p*-Value
	CoG (*n* = 18)	SafG (*n* = 19)	ChG (*n* = 19)	PG (*n* = 19)	
**Variables**	Mean ± SD	Mean ± SD	Mean ± SD	Mean ± SD	
**Energy (Kcal)**					
T1	2148.88 ±126.51	2080.81 ±122.59	2094.59 ± 94.23	2070.64 ± 109.96	
T2	1536.19 ± 78.60 *	1580.32 ± 98.69 *	1562 ± 68.66 *	1538.14 ± 63.82 *	
**Δ**	−612.69 ± 81.2 **	**−500.49 ± 73.54**	**−532.59 ± 75.38**	**−532.50 ± 89.84**	***p* = 0.016**
**Protein (g)**					
T1	59.71 ± 6.39	57.81 ± 5.79	62.31 ± 8.65	58.68 ± 6.46	
T2	62.84 ± 4.58 *	60.86 ± 4.96 *	63.19 ± 3.87	61.62 ± 3.78	
Δ	**3.14 ± 7.56**	**3.04 ± 5.75**	**0.87 ± 8.15**	**2.95 ± 7.33**	***p* = 0.735**
**Carbohydrates (g)**					
T1	354.79 ± 42.66	349.26 ± 24.23	352.42 ± 18.76	345.70 ± 24.70	
T2	216.24 ± 14.83 *	220.28 ± 15.31 *	223.07 ± 12.03 *	217.98 ± 14.32 *	
**Δ**	**−138.55 ± 41.19**	**−128.98 ± 20.00**	**−129.34 ± 17.56**	**−127.71 ± 28.19**	***p* = 0.375**
**Lipids (g)**					
T1	54.55 ± 11.11	50.28 ± 7.18	48.41 ± 6.68	50.35 ± 6.91	
T2	51.65 ± 3.81	48.64 ± 4.26	46.33 ± 3.59	48.63 ± 3.28 *	
**Δ**	**−2.9 ± 6.21**	**1−1.69 ± 5.89**	**−2.08 ± 5.33**	**−1.71 ± 6.44**	***p* = 0.116**
**Fiber (g)**					
T1	11.48 ± 1.98	12.14 ± 1.84	12.36 ± 1.50	12.20 ± 1.68	
T2	25.94 ± 2.86 *	25.71 ±2.26 *	24.63 ± 3.07 *	26.28 ± 2.86 *	
**Δ**	**14.46 ± 3.59**	**13.57 ± 2.56**	**12.26 ± 2.44**	**14.08 ± 2.18**	***p* = 0.109**

Data given as means and standard deviation (SD). *p* < 0.05 indicates a different significance: * = T1 vs. T2; ** = CCo vs. GCh, GCA, and GP. CoG—Group supplemented with coconut oil; SafG—Group supplemented with safflower oil; ChG—Group supplemented with chia oil; PG—Group supplemented with soybean (placebo) oil. Δ = T1 − T2.

**Table 5:** Level of physical activity of the participants before (T1) and after (T2) the study.

Classification	Groups										*p*-Value
	CoG (*n* = 18)		SafG (*n* = 19)		ChG (*n* = 19)		PG (*n* = 19)		Total		
	*n*	%	*n*	%	*n*	%	*n*	%	*n*	%	
Total	18	100.0	19	100.0	19	100.0	19	100.0	75	100.0	
**T1**											*p* = 0.031 *
Sedentary	14	77.8	15	78.9	16	84.2	19	100.0	63	84.0	
Active	4	22.2	4	32.1	3	15.8	-	-	12	16.0	
**T2**											*p* = 0.278
Sedentary	7	38.9	6	31.6	5	26.3	19	47.4	45	60.0	
Active	11	61.1	13	68.4	14	73.7	10	52.6	30	40.0	
***p*-value**	***p* = 0.044**		***p* = 0.041**		***p* = 0.021**		**		***p*** < 0.001 *		

Subjective evaluation of the participants’ physical activity before (start) and after (last) the study period. * = *p* < 0.05. ** = Not determined due to the difference in the number of response categories between the two evaluations. CoG—Group supplemented with coconut oil; SafG—Group supplemented with safflower oil; ChG—Group supplemented with chia oil; PG—Group supplemented with soybean (placebo) oil.

## Supplementary Materials

The following are available online at http://www.mdpi.com/2072-6643/10/7/932/s1, Table S1: Centesimal Composition of the oils used in the study. Table S2: Description of anthropometric and biochemical evaluations.
