# Supplementary material for: Supplementation-Dependent Effects of Vegetable Oils with Varying Fatty Acid Compositions on Anthropometric and Biochemical Parameters in Obese Women
Source: Nutrients. 2018 Jul 20;10(7):932. doi: 10.3390/nu10070932 (PMC6073593; doi:10.3390/nu10070932)
Supplement: Supplementary file 1 [file nutrients-10-00932-s001.pdf]

Supplementary file 1

Table 1 - Centesimal composition of the Oils used in this study.

FATTY ACIDS	Composition (%)			
	Coconut oil[a]	Safflower oil [b]	Chia oil[c]	Soybean oil[d]
Oléic (C18:1)	4,73	13,34	9,83	24,33
Linoleic (C18:2)	2,15	81,15	9,98	48,32
Linolenic (C18:3)	---	0,7	66,57	7,16
Palmitic (C16:0)	8,65	2,48	8,56	14,1
Estearic (C18:0)	2,05	2,33	2,88	4,78
Miristic (C14:0)	17,27	----	1,34	1,11
Caprylic acid (C8:0)	5,3	----	--	--
Capric (C10:0)	4,72	----	--	--
Lauric (C12:0)	55,13	----	--	--
Eicosanoic (C20:0)	----	----	0,84	0,19
TOTAL	100%	100%	100%	100%

Source: Food Analysis Laboratory - Federal University of Pernambuco. Chromatography Laboratory - Department of Chemical Engineering - Federal University of Pernambuco. [a]Cocos nucifera L. [b] Carthamus tinctorius.[c]Salvia Hispanica [d] Óleo de soja

Table 2 - Description of anthropometric and biochemical evaluations.

Anthropometric and biochemical assessments used in the study	
Weight (Kg)	The participant was placed in the center of the base of a calibrated platform weighing 150 kg and divided into 100 g (WHO, 2008)[1].
Height (cm)	The participant stood barefoot, her heels together, her back straight, her arms extended along her body. The height was measured through a 2 meter stadiometer

	divided in centimeters (Lohman et al 1988)[2].
Body mass index(Kg/m^2)	It was calculated from the formula: Current weight in kg / (Height in m)^2 (Garrow & Webster, 1985)[3].
Waist circumference (cm)	It was measured with the volunteer standing with a tape measure at the midpoint between the iliac crest and the last rib (WHO, 2008)
Waist Height Ratio	It was calculated from the formula: WC in cm / Height in cm. (Haum et al., 2009).
Conicity index	It was calculated from formula (Valdez, 1991) $\text{conicity index} = \frac{\text{waist circumference (m)}}{0.109\sqrt{\text{weight (kg)}\backslash \text{stature (m)}}}$
Total Cholesterol (TC)	Automated enzymatic method
Low-density lipoprotein cholesterol	Automated enzymatic method
**High-density lipoprotein cholesterol** (HDLc)	Automated enzymatic method
Triglycerides	Automated enzymatic method
Hemoglobina glicosilada (HbA1c)	High performance liquid chromatography (HPLC).
Estimated Mean Glycemia	GME = 28,7 × Hb A1c – 46,7 (Nathan et al, 2008)
